# Development and Optimisation of an HPLC-DAD-ESI-Q-ToF Method for the Determination of Phenolic Acids and Derivatives

**DOI:** 10.1371/journal.pone.0088762

**Published:** 2014-02-14

**Authors:** Annalaura Restivo, Ilaria Degano, Erika Ribechini, Maria Perla Colombini

**Affiliations:** Dipartimento di Chimica e Chimica Industriale, Università di Pisa, Pisa, Italy; Deutsches Krebsforschungszentrum, Germany

## Abstract

A method for the HPLC-MS/MS analysis of phenols, including phenolic acids and naphtoquinones, using an amide-embedded phase column was developed and compared to the literature methods based on classical C18 stationary phase columns. RP-Amide is a recently developed polar embedded stationary phase, whose wetting properties mean that up to 100% water can be used as an eluent. The increased retention and selectivity for polar compounds and the possibility of working in 100% water conditions make this column particularly interesting for the HPLC analysis of phenolic acids and derivatives. In this study, the chromatographic separation was optimised on an HPLC-DAD, and was used to separate 13 standard phenolic acids and derivatives. The method was validated on an HPLC-ESI-Q-ToF. The acquisition was performed in negative polarity and MS/MS target mode. Ionisation conditions and acquisition parameters for the Q-ToF detector were investigated by working on collision energies and fragmentor potentials. The performance of the method was fully evaluated on standards. Moreover, several raw materials containing phenols were analysed: walnut, gall, wine, malbec grape, French oak, red henna and propolis. Our method allowed us to characterize the phenolic composition in a wide range of matrices and to highlight possible matrix effects.

## Introduction

Natural phenols are classified as natural organic substances, featuring one or more phenolic groups in their structure. These aromatic compounds are the main group of secondary metabolites and bioactive substances in plants, and are also widespread in the microorganism kingdom. Secondary metabolites play various roles in plant metabolism, such as growth, photosynthesis and reproduction.

Phenols are also important in terms of their antioxidant activity: they are known to react with free superoxide radicals, thus protecting against oxidative processes. Natural phenols are thus widely employed in the agricultural, biological, chemical and pharmaceutical fields [1], [2]. Due to this antioxidant activity along with the impact on the human metabolism, natural phenols have been extensively studied. Several analytical techniques are currently used for identifying and quantifying these compounds in a wide range of matrices. Depending on the target of the study, bulk analysis is performed by spectrophotometric assays [3], [4], [5], NMR [4] or TLC [6]. Natural phenols include a large variety of substances, often found as complex mixtures. For this reason, the most common analytical methods used for their analysis are based on separative techniques, such as capillary electrophoresis [4], gas chromatography and high performance liquid chromatography (HPLC) [4], [5], [7], [8], [9]. HPLC using reverse phase C18 columns is the most commonly used method given its high polarity and solubility in most common eluents [4], [7], [9], [10]. In addition, phenols have strong UV absorbance and the most commonly used detectors for liquid chromatography are UV-Vis [3], [7], [11].

Despite this, the resolution and sensitivity of currently employed HPLC-DAD methods can be further improved. HPLC coupling with a mass spectrometer detector enhances selectivity and specificity [4], [9], [10], [12]. In addition, the use of chromatographic columns embedded with stationary phases seems to provide better resolution by improving chromatographic separation [13], [14].

RP-Amide is a recently developed polar embedded stationary phase, whose wetting properties mean that 100% water can be used as an eluent. RP-Amide shows increased dipole-type interactions and higher interactions with lone pair and π-electrons donor solutes. These properties increase in the retention and selectivity for polar compounds, compared to classical C18 columns [15], [16].

This paper deals with the development of an analytical procedure for the determination of two particular classes of natural phenols: phenolic acids and naphthoquinones. Our interest in these molecules is due not only to their physiological role in human and plant metabolism, but also to their importance in the food industry (wine, honey) and their use in textile dyeing. These molecules are the main constituents of several natural raw materials commonly used in the past for dyeing purposes, for preparing inks and for tanning leather [17], [18].

We developed and optimized an HPLC-DAD-ESI-Q-ToF method for the analysis of 13 phenolic acids and derivatives, including hydroxybenzoic acids, hydroxycinnamic acids and naphthoquinones.

First, we describe how we optimized the chromatographic separation, using an HPLC-DAD with an amide-embedded phase column. We tested the performances of the RP-Amide column for the analysis of phenolic acids and naphtoquinones and its advantages were highlighted by comparing results obtained with the chromatograms obtained using a classical C18 stationary phase. Second, we describe the optimization of ESI-Q-ToF detection. Finally, we show the method was validated by evaluating the resolution, linearity, sensitivity and precision thus highlighting that our method is appropriate for the detection of these classes of compounds in complex mixtures. Moreover, several raw materials containing phenols were analysed. The phenolic composition of a wide range of matrices (walnut, gall, wine, malbec grape, French oak, red henna and propolis) was thus characterised, proving the suitability of the method in terms of sensitivity, separative performances and reproducibility.

## Materials and Methods

### 1. Chemicals

Acetonitrile, water and methanol (LC-MS Chromasolv grade, >99.9% purity) used for sample pre-treatment and as HPLC-ESI-MS eluents were from Fluka (Milan, Italy). Eluents for HPLC-DAD analysis were acetonitrile (Chromasolv for HPLC, >99.8%) from Sigma Aldrich (Milan, Italy) and water (RPE) from Carlo Erba. Formic acid 98% purity was from J.T. Baker. Gallic acid (more than 99% purity) and ellagic acid dehydrate (97% purity) were from Alpha Aesar (Lancaster, England), 4-hydroxybenzoic acid (99% purity), 3,4-dihydroxybenzoic acid (>97% purity), 2,4-dihydroxybenzoic acid (97% purity), dihydrocaffeic acid (98% purity), ferulic acid (99% purity), syringic acid (98% purity), vanillic acid (97% purity), caffeic acid (97% purity), juglone (97% purity) and lawsone (97% purity) were from Sigma Aldrich (Milan, Italy).

The stock solution contained 13 natural phenols in methanol (100 µg/g). Aliquots of the stock solution were diluted with water to obtain working standards for method development. The HPLC-DAD requires a standard concentration in the range of 10 µg/g, while HPLC-ESI-Q-ToF of 3 µg/g. Solutions were stored at −18°C.

### 2. Raw Materials

Gall, walnut and red henna were from Kremer Pigmente (Aichstetten, Germany). Wine was a “Rosso di Montalcino”, Sangiovese grape (Cantina di Montalcino, Italy). Malbec grape and French oak were from a local reseller of enological products. Propolis was kindly provided by a local beekeeper.

### 3. Instruments and Working Conditions

HPLC-DAD analyses were performed with an HPLC quaternary pump PU2089 (Jasco int.) equipped with a degasser, an injection valve Rheodyne (USA), and a 20 µL capacity loop. The pump was also coupled with a diode array MS-2010 detector (Jasco int., Japan). The detector operated in the range of 200 and 650 mm, with a 4 nm resolution.

Two set-ups were tested and compared:

Separation was performed on a TC-C18 reverse phase column 250 × 4.6 mm, particle size 5 µm (Agilent Technologies, Palo Alto, CA, USA). Eluents were water (A) and acetonitrile (B), both with 0.3% formic acid [19], [20], [21], [22], [23] and the flow rate was set at 1 mL/min. The gradient was linear: 0–5 minutes, 95% A; 5–20 min, from 5% to 15% B; 20–35 min, from 15% to 20% B; 35–50 min, from 20 to 70% B; 50–52 min, from 70% to 100% B.
Separation was performed on an Ascentis Express RP-Amide column 100 x 2.1 mm, particle size 2.7 µm (Supelco, Sigma Aldrich, Milan, Italy). Eluents were water (A1) and acetonitrile (B1) with 1.4% formic acid (FA) and the flow rate was set at 0.3 mL/min. The gradient was linear: 0–5 minutes, 100% A1; 5–10 min, from 0% to 4% B1; 10–32 min, from 4% to 30% B1; 32–44 min, from 30% to 100% B1.

HPLC-ESI-Q-ToF analyses were performed with an HPLC 1200 Infinity (Agilent Technologies, Palo Alto, CA, USA) coupled to a Jet Stream ESI-Q-ToF 6530 Infinity (Agilent Technologies, Palo Alto, CA, USA). Injection volume was set at 2 µL. Column temperature was 30°C. Separation was performed on the Ascentis Express RP-Amide column (set-up B). ESI operation conditions were: drying gas (N_2_, purity >98%) temperature 350°C, drying gas flow 10 L/min, nebulizer gas pressure 35 psig, sheath gas (N_2_, purity >98%) temperature 375°C, sheath gas flow 11 L/min, capillary voltage 4.5 KV. High resolution MS and MS/MS acquisition range was set from 100 to 1700 m/z. Nozzle, skimmer and octapole RF voltages were set at 1000 V, 65 V and 750 V, respectively. After optimization, the declustering potential was set at 150 V in the time segment between 0 and 26.5 minutes, while after 26.5 min was set at 175 V.

Collision gas for MS/MS analysis was nitrogen (purity 99.999%). Data were collected by target MS/MS acquisition with an MS and MS/MS scan rate of 1.41 spectra/sec. The masses selected for MS/MS analysis after optimization are reported in Table 1, along with the selected collision energies for each molecular transition.

**Table 1:** MS/MS parameters (*The product ion was selected during post-processing of the data for the quantitation of the analytes).

Analyte	Precursor ion [M-H]^−^	CE (V)	Product ion MS/MS(*)
Gallic acid	169.014	20	125.025
3,4 dihydroxybenzoic acid	153.019	20	109.029
Dihydrocaffeic acid	181.051	–	–
4-hydroxybenzoic acid	137.024	–	–
Vanillic acid	167.035	–	–
2,3-dihydroxybenzoic acid	153.019	20	109.029
Syringic acid	197.046	–	–
Caffeic acid	179.035	20	135.045
2,4 dihydroxybenzoic acid	153.019	20	109.029
Ferulic acid	193.051	–	134.037
Lawsone (2-hydroxy-1,4-naphthoquinone)	173.024	20	145.030
Ellagic acid	300.999	10	175.039
Juglone (5-hydroxy-1,4-naphthoquinone)	173.024	20	145.030

The mass axis was calibrated using the Agilent tuning mix HP0321 (Agilent technologies) prepared in acetonitrile. Mass spectrometer control, data acquisition and data analysis were performed with MassHunter® Workstation software (B.04.00).

### 4. Method Validation

The method was evaluated on the basis of system suitability, linearity, sensitivity, and repeatability.

Retention time, retention factor and selectivity were tested to check the system suitability of the HPLC-ESI-Q-ToF method developed. Method linearity was tested on the basis of calibration curves, which were processed using linear regression. Five standard solutions were prepared as water dilutions of the stock standard solution from 0.10 to 4.00 µg/g, and were used for calibration. For lawsone and ellagic acid, two additional standard solutions at 0.02 and 0.05 µg/g were analysed.

Method sensitivity was evaluated by testing detection (LOD) and quantitation (LOQ) limits. Validation was performed using the lowest concentration level giving a visible signal with the HPLC-ESI-Q-ToF. Six replicates of these standard solutions were analysed and standard deviations of chromatographic areas were used for LOD and LOQ calculation. LOD values were assigned as the blank average value plus three times the standard deviation of the analytes signal. LOQ values were calculated as the blank average value plus 10 times the standard deviation of the analytes signal.

The repeatability of the method was evaluated by checking intraday and interday precision. Precision was evaluated by analysing three replicates of each point of the calibration curve, in the same day (intraday precision) and in over several days (interday precision).

## Results and Discussion

### 1. Optimization of Chromatographic Separation

**Figure 1:**
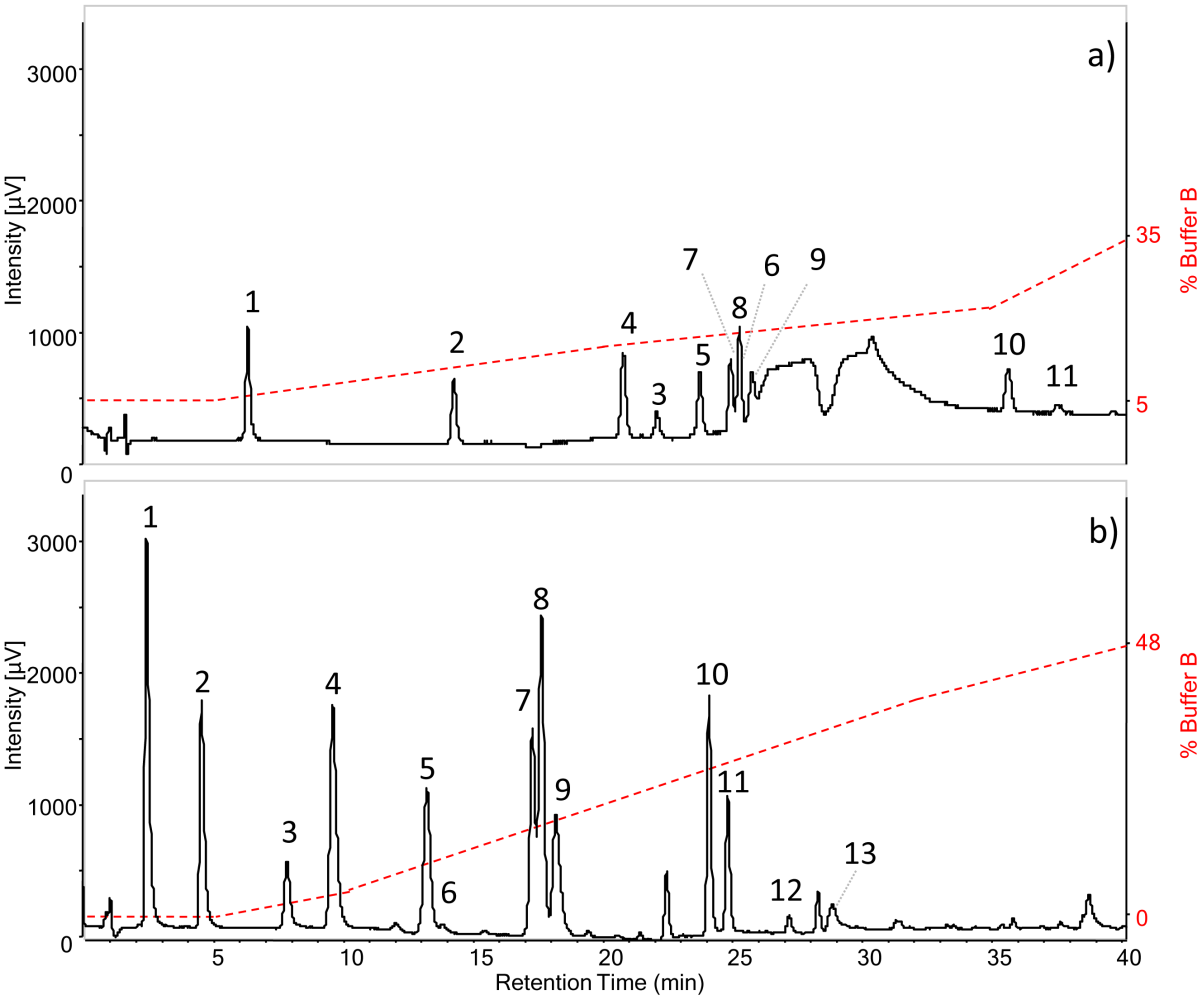
HPLC-DAD chromatograms at 275 nm of standard phenols solution analysed with: (a) C18 reverse phase column and eluents H_2_O (0.3% FA) and ACN (0.3% FA), flow rate: 1 mL/min; (b) RP-Amide column after gradient optimization with eluents H_2_O (1.4% FA) and ACN (1.4% FA), flow rate: 0.3 mL/min. Both gradients are described in Materials and methods, paragraph 2.3. The analytes are: gallic acid (1), 3,4-dihydroxybenzoic acid (2), dihydrocaffeic acid (3), 4-hydroxybenzoic acid (4), vanillic acid (5), 2,3-dihydroxybenzoic acid (6), caffeic acid (7), syringic acid (8), 2,4-dihydroxybenzoic acid (9), ferulic acid (10), lawsone (11), juglone (12), ellagic acid (13).

The target analytes include hydroxybenzoic acids, hydroxycinnamic acids and naphthoquinones. The HPLC-DAD method was developed by setting up of a chromatographic gradient to obtain a complete separation of the 13 phenolic acids and derivatives. To take full advantage of the column properties, the gradient employed starts from 1.4% formic acid in water and ends with 1.4% formic acid in acetonitrile, as described in the Materials and Methods. The results were compared to the same analysis performed on a classical reverse phase C18 column. The two columns differ completely in terms of retention, as can be seen in the chromatograms in Figure 1. The RP-Amide column separates several peaks which almost co-elute with classical set-up. Juglone and ellagic acid were not detected after injection on the C18, whereas RP-Amide managed to separate and detect all the analytes in the standard mixture.

### 2. Optimization of Detection with ESI-Q-ToF

Detection parameters such as selectivity and sensitivity were optimized on the HPLC-ESI-Q-ToF. On the basis of literature data and preliminary tests, a negative ionization mode was chosen. pH conditions, fragmentor potential (also known as declustering potential) and MS/MS parameters were tested during the method development.

#### 2.2. pH conditions

**Figure 2:**
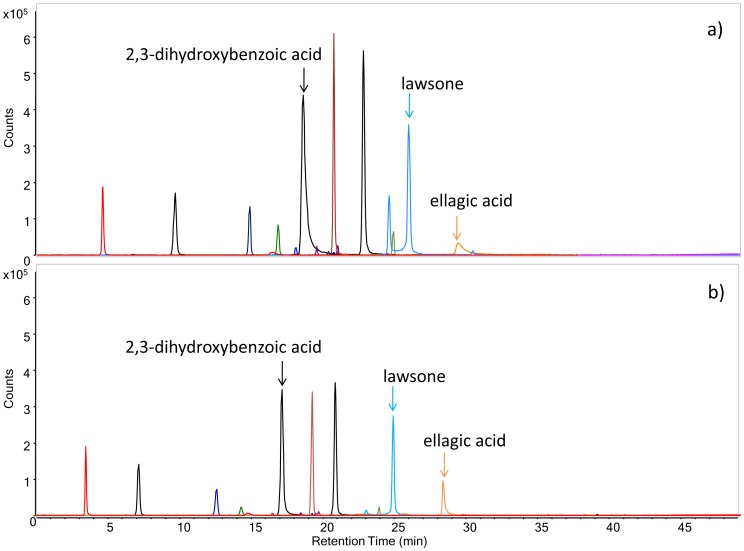
HPLC-ESI-MS extracted ion chromatograms of phenol standard mixture eluted with acetonitrile/water with 0.3% (a) and 1.4% (b) of formic acid. The gradient and ESI parameters are described in Materials and methods, paragraph 2.3. The extracted ions correspond to the pseudo-molecular ions of the analytes (see Table 1).

Several concentrations of the mobile phase modifier (formic acid) were tested while developing the HPLC-ESI-Q-ToF method, due to the sensitivity of the ion source to the pH of the eluents. Experiments were performed using various combinations of water and acetonitrile in the range between 0.3% and 1.4% formic acid (FA). As expected, pH influenced both ionization and retention, with a consequent modification in peak intensity and resolution, as demonstrated by the examples given in Figure 2. Specifically, lower pH conditions (1.4% FA, pH = 2.1) yielded an increase in resolution and symmetry of the peaks, especially for 2,3-dihydroxybenzoic acid, lawsone and ellagic acid (Figure 2b), while at a higher pH (0.3% FA, pH = 2.4), the intensity of the peaks increases (Figure 2a). In order to obtain the highest resolution, we added 1.4% formic acid to both the eluents in the optimized, final method.

#### 2.3. Declustering potential

**Figure 3:**
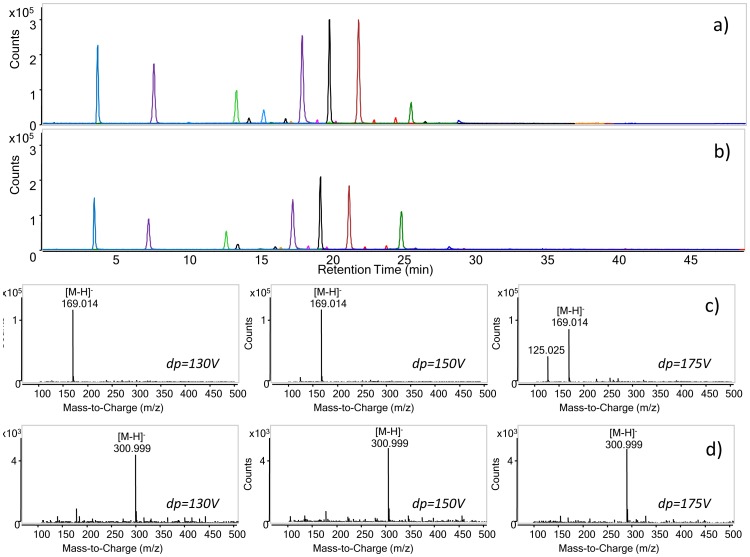
HPLC-ESI-MS extracted ion chromatograms of phenol standard mixture analysed at 150 V (a) and 175 V (b) declustering potential (dp). The gradient and ESI parameters are described in Materials and methods, paragraph 2.3. The extracted ions correspond to the pseudo-molecular ions of the analytes (see Table 1). Mass spectra of gallic (c) and ellagic acid (d) at different values of declustering potential.

Three declustering potential values were tested: 130 V, 150 V (chromatogram in Figure 3a) and 175 V (chromatogram reported in Figure 3b). The main ion detected for each analyte was the pseudo-molecular ion [M–H]^−^. The application of a declustering potential of 130 V resulted in a lower intensity of the [M–H]^−^ ion with respect to the other potentials, while 175 V resulted in slightly higher intensities. In some cases (see gallic acid mass spectrum in Figure 3c), the application of 175 V resulted in a fragmentation of the pseudo-molecular ion; thus, an intermediate declustering potential of 150 V was selected for some analytes. On the basis of the results, the HPLC-MS acquisition method was divided into two time segments for the analysis of juglone, lawsone and ellagic acid: in the first part of the run the declustering potential was set at 150 V, while in the second at 175 V.

#### 2.4. MS/MS analysis

**Figure 4:**
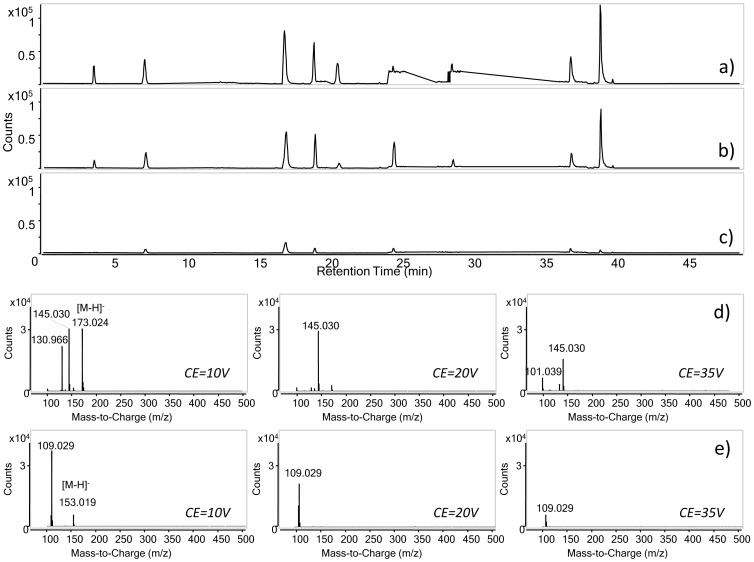
HPLC-ESI-MS/MS chromatograms of phenol standard mixture analysed with collision energy (CE) 10 V (a), 20 V (b), and 35 V (c). The gradient and ESI parameters are described in Materials and methods, paragraph 2.3, as well as acquisition parameters in MS/MS mode (see Table 1). Tandem mass spectra of lawsone (d) and 3,4-dihydroxybenzoic acid (e) obtained at different collision energies.

MS/MS detection for each analyte was optimised by selecting precursor/product ions and working on collision energy values for MS/MS target analysis. Table 1 lists the m/z values selected for each analyte. Collision energies for MS/MS analysis were tested in the range from 4 to 35 V. HPLC-ESI-MS/MS chromatograms of phenol standard mixture analysed with collision energy (CE) 10 V, 20 V, and 35 V are presented in Figure 4 a, b and c, respectively. The mass spectra of lawsone and spectra 3,4-dihydroxybenzoic acid are presented as examples in Figure 4d and e.

**Figure 5:**
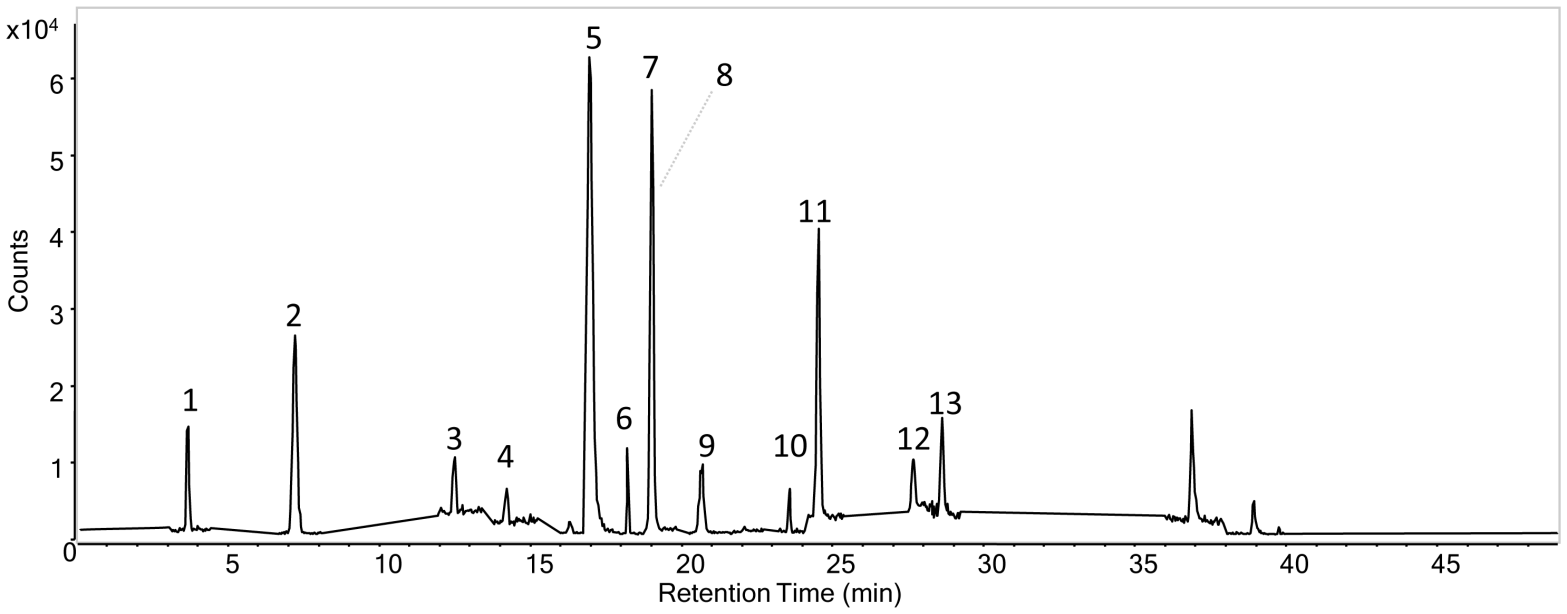
HPLC-ESI- MS/MS chromatogram of the mixture of 13 phenolic acids and derivatives analysed with the RP-Amide column after optimization. Chromatographic and detection parameters are detailed in paragraph 2.3. The analytes are: gallic acid (1), 3,4-dihydroxybenzoic acid (2), dihydrocaffeic acid (3), 4-hydroxybenzoic acid (4), vanillic acid (5), 2,3-dihydroxybenzoic acid (6), caffeic acid (7), syringic acid (8), 2,4-dihydroxybenzoic acid (9), ferulic acid (10), lawsone (11), juglone (12), ellagic acid (13).

The result of the chromatographic separation and detection of the standard mixture obtained with the optimized HPLC-ESI-Q-ToF method is shown in Figure 5.

### 3. Method Validation

The performance of the method was evaluated on the basis of system suitability, linearity, sensitivity, and repeatability. The results are reported in Tables 2 and 3.

**Table 2:** System suitability parameters.

Analyte	Retention time (Rt)		Retention Factor (k’)		Selectivity (α)		Theoretical plates (N)	
	C18	RP-Amide	C18	RP-Amide	C18	RP-Amide	C18	RP-Amide
Gallic acid	8.46	3.56	1.00	3.47	2.69	2.32	16000	6000
3,4-dihydroxybenzoic acid	15.58	7.20	2.67	8.04	1.51	1.84	43000	13000
Dihydrocaffeic acid	22.59	12.60	4.33	14.83	1.08	1.14	101000	50000
4-hydroxybenzoic acid	21.41	14.28	4.05	16.93	1.07	1.16	81000	66000
Vanillic acid	24.06	16.48	4.67	19.71	1.05	1.04	108000	97000
2,3-dihydroxybenzoic acid	26.15	17.11	5.17	20.50	1.39	1.08	98000	45000
Syringic acid	25.43	18.50	5.00	22.24	1.02	1.04	141000	171000
Caffeic acid	25.12	19.27	4.92	23.20	1.01	1.08	131000	118000
2,4-dihydroxybenzoic acid	25.86	20.82	5.10	25.16	1.01	1.16	122000	80000
Ferulic acid	34.70	23.95	7.18	29.08	1.06	1.04	120000	274000
Lawsone (2-hydroxy-1,4-naphthoquinone)	36.43	24.90	7.59	30.28	–	1.15	141000	149000
Ellagic acid	–	28.41	–	34.68	–	1.02	–	171000
Juglone (5-hydroxy-1,4-naphthoquinone)	–	29.05	–	35.49	–	–	–	82000

**Table 3:** Validation parameters.

Standard	Slope (Area*vs* ng/g)	Intercept(Area)	R^2^	LOD (µg/g)	LOQ (µg/g)	RDS intraday	RDS interday
Gallic acid	41.4	2070	0.9997	0.15	0.50	3.5	5.3
3,4-dihydroxy benzoic acid	202	−619	0.9994	0.01	0.05	1.2	3.0
Dihydrocaffeic acid	80.0	5200	0.9933	0.03	0.11	3.3	3.5
4-hydroxybenzoic acid	41.0	−2240	0.9997	0.04	0.14	1.1	4.8
Vanillic acid	8.55	−110	0.9987	0.01	0.03	0.9	10.7
2,3-dihydroxybenzoic acid	479.0	22600	0.9997	0.01	0.03	2.2	5.9
Syringic acid	4.70	−203	0.9781	0.01	0.02	7.6	14.7
Caffeic acid	322	1970	0.9999	0.01	0.02	1.6	3.1
2,4-dihydroxy benzoic acid	50.8	19.3	0.9955	0.03	0.08	12.6	12.9
Ferulic acid	6.12	−197	0.9771	0.01	0.04	10.6	13.5
Lawsone(2-hydroxy-1,4-naphthoquinone )	867	−3330	0.9977	0.01	0.03	4.9	19.5
Ellagic acid	386	688	0.9982	0.02	0.06	9.5	15.7
Juglone(5-hydroxy-1,4-naphthoquinone)	52.0	−9318	0.9984	0.01	0.02	3.9	13.3

#### 3.1. System suitability

In detail, retention time, retention factor and selectivity were tested to check the system suitability of the HPLC-ESI-Q-ToF method developed. Table 2 presents the values obtained with the optimized method, compared to the results obtained employing the C18 column. The analysis of phenolic acids and derivatives with an RP-Amide column gives a sufficient resolution, enabling all the target analytes to be separated.

#### 3.2. Linearity

Method linearity was tested on the basis of calibration curves, processed using linear regression. Five standard solutions, prepared as water dilutions of the stock standard solution from 0.1 to 4 µg/g, were used for calibration. For lawsone and ellagic acids, two more standard solutions at 0.02 and 0.05 µg/g were analysed. Correlation coefficients of each analyte were above 0.99, showing a good linearity. Only syringic and ferulic acids showed lower correlation coefficients, of around 0.97 (Table 3).

#### 3.3. Sensitivity

Method sensitivity was evaluated by testing detection (LOD) and quantitation (LOQ) limits. Validation was performed using the lowest concentration level giving a visible signal with HPLC-ESI-Q-ToF instrumentation. Six replicates of these standard solutions were analysed and the standard deviations of chromatographic areas was used for LOD and LOQ calculation.

Table 3 reports the values obtained, ranging between 0.01 and 0.15 µg/g for LOD and 0.02 and 0.50 µg/g for LOQ. These values are lower than the ones described in the literature (0.3 µg/g for LOD and 0.1 µg/g for LOQ) for phenols with an HPLC-DAD equipped with an amide-embedded phase [13].

#### 3.4. Repeatability

The repeatability of the method was evaluated by checking intraday and interday precision. Precision was evaluated by analysing three replicates of each point of the calibration curve, in the same day (intraday precision) and in between several days (interday precision). The relative standard deviation (RSD) of replicates was calculated (Table 3). For all analytes, intraday RSD values were below 10% and interday RSDs were below 20%.

### 4. Application to Complex Matrices

**Figure 6:**
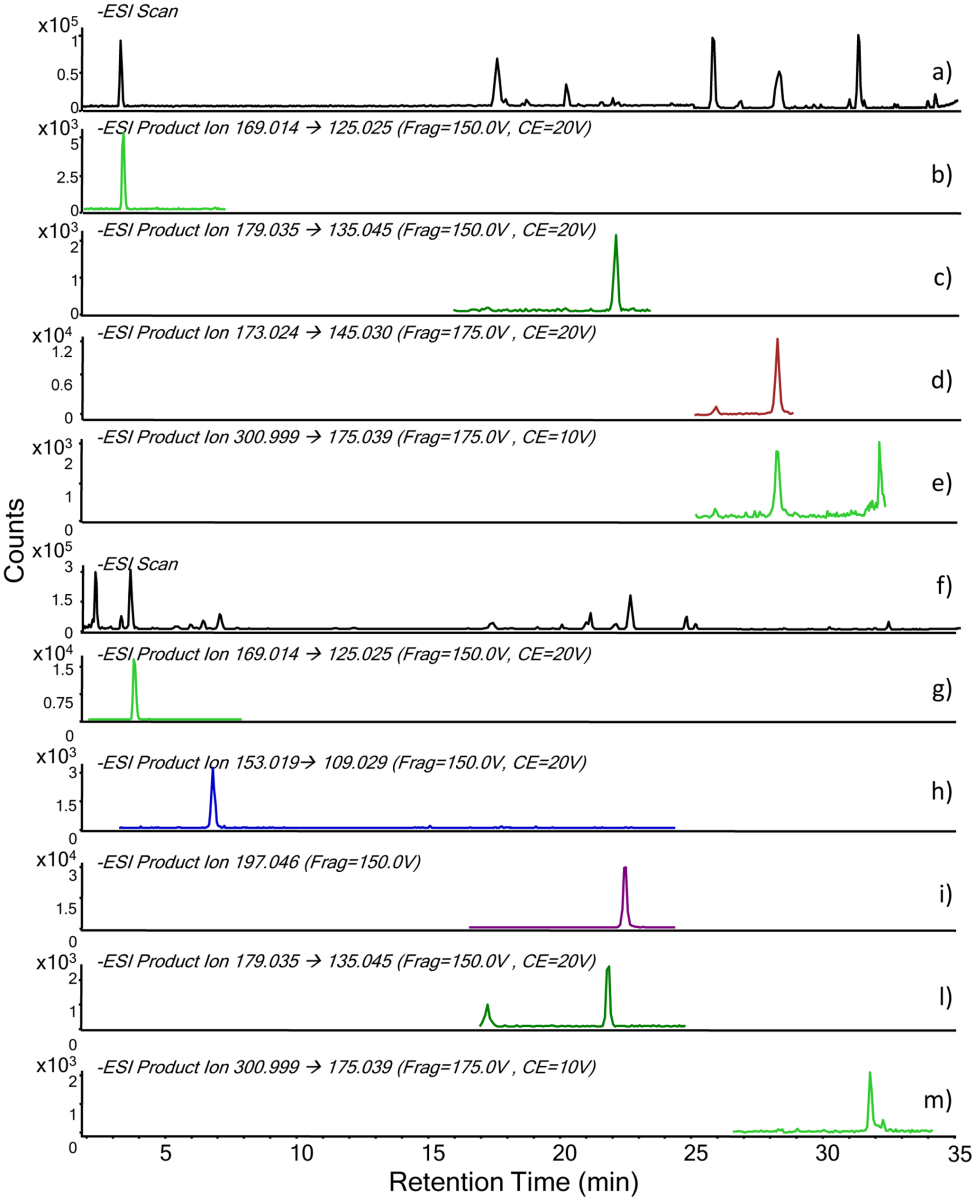
HPLC-MS-ESI-Q-ToF chromatograms. Red henna: (a) Full scan; (b) MS/MS chromatogram of gallic acid; (c) MS/MS chromatogram of caffeic acid; (d) MS/MS chromatogram of lawsone; (e) MS/MS chromatogram of ellagic acid. Wine: (f) Full scan; (g) MS/MS chromatogram of gallic acid; (h) MS/MS chromatogram of 3,4-dihydroxybenzoic acid: (i) MS/MS chromatogram of syringic acid; (l) MS/MS chromatogram of caffeic acid; (m) MS/MS chromatogram of ellagic acid.

In order to test the method’s applicability to complex matrices, several raw materials containing phenols were analysed: walnut, gall, wine, malbec grape, French oak, red henna and propolis. Our method allowed us to characterize the phenolic composition in a wide range of matrices and the results are reported in Table 4. Gallic acid, 3,4-dihydroxybenzoic acid, 4-hydroxybenzoic acid, syringic acid, caffeic acid, ferulic acid, lawsone and ellagic acid were identified in one or more samples. Positive and negative matrix effects, known to affect HPLC-ESI-Q-ToF analysis [24], were observed and described for several analytes. Figure 6 shows the chromatograms obtained from the HPLC-ESI-Q-ToF analysis of henna and wine, and in particular the extracted ion MS/MS chromatograms of each analyte investigated. The analysis of samples spiked with suitable standard solutions of polyphenols allowed us to highlight the occurrence of matrix effects due to the complexity of the analysed extracts. In particular, both positive and negative matrix effects were determined. In the majority of cases, positive effects were evidenced, thus suggesting the presence of matrix components that cause an increase in the ionization yield of the target analytes. On the contrary, no effect on the separation of the analytes was observed, thus confirming the suitability of the chromatographic set-up for the characterisation of the extracts of complex natural substances.

**Table 4:** Analytes identified in the matrices investigated. (+) and (−) indicate the presence of a matrix effect respectively positive and negative.

Matrix	Analytes
Walnut	Gallic acid (−), 3,4-dihydroxybenzoic acid (+), 4-hydroxybenzoic acid (−), ellagic acid (+)
Gall	Gallic acid (+), 3,4-dihydroxybenzoic acid (+), syringic acid (+), ellagic acid (+)
Wine	Gallic acid, 3,4-dihydroxybenzoic acid (+), syringic acid, caffeic acid (+), ellagic acid (+)
Malbec grape	Gallic acid, 3,4-dihydroxybenzoic acid (+), ellagic acid (+)
French oak	Gallic acid, 3,4-dihydroxybenzoic acid (+), ellagic acid (+)
Red henna	Gallic acid (+), caffeic acid (+), lawsone (−), ellagic acid (+)
Propolis	Gallic acid (+), 3,4-dihydroxybenzoic acid (+), caffeic acid (+), ferulic acid (+), ellagic acid (+)

## Conclusions

Thanks to the use of an innovative chromatographic column coupled with mass spectrometric detection, our method for analysing phenolic acids and derivatives significantly increases selectivity and sensitivity with respect to the current literature.

The specific advantages of the RP-Amide column in phenolic acids and derivatives analysis were highlighted. This column, used with a water/acetonitrile gradient and formic acid buffer, enabled us to work in 100% water conditions and provided an increase in separation selectivity.

The HPLC-ESI-Q-ToF further increased the selectivity and sensitivity of our method. Negative ionization mode was selected based on literature data and preliminary tests. Optimized conditions for MS and target MS/MS detection, obtained by setting specific values of declustering potential and collision energies for each analyte, led to the identification and quantification of 13 different phenolic acids and naphtoquinones. The method was validated through the evaluation of resolution, linearity, sensitivity and precision.

This fully validated method was further tested for the qualitative analysis of phenolic acids and derivatives in several complex matrices, such as walnut, gall, wine, malbec grape, French oak, red henna and propolis. The phenolic composition in this wide range of matrices was quantified and possible matrix effects were highlighted.
